# The Personal Beliefs About Aging Scale

**DOI:** 10.1093/geroni/igaa057.1726

**Published:** 2020-12-16

**Authors:** Lauren Bowen, Nina Silverstein, Susan Whitbourne, Taylor Jansen

**Affiliations:** 1 University of Massachusetts, Boston, Boston, Massachusetts, United States; 2 University of Massachusetts Boston, Needham, Massachusetts, United States; 3 University of Massachusetts Amherst, Boston, Massachusetts, United States; 4 University of Massachusetts Boston, Boston, Massachusetts, United States

## Abstract

Most existing ageism scales (e.g., Fabroni et al., 1990; Laidlaw et al., 2007; Rupp et al., 2005) are designed to measure younger people’s age attitudes, often asking respondents to affirm or reject stereotypes of older people. The Personal Beliefs about Aging (PBA) Scale gathers information about the degree to which respondents, regardless of age, value age diversity on their campus. Preliminary findings from a 2019-2020 University of Massachusetts system-wide electronic survey that yielded 2,563 responses across 3 (of 5) campuses indicate that most faculty (83%), staff (84%), and students (72%) perceived ageism as a serious problem in society; however far fewer considered ageism as a serious problem on their own campus, with students (20%) perceiving campus ageism to an even lesser extent than faculty (39%) and staff (36%). Part of a symposium sponsored by Age-Friendly University (AFU) Interest Group.

